# Development and evaluation of an internet-based blended-learning module in biomedicine for university applicants – Education as a challenge for the future –

**DOI:** 10.1186/s13005-016-0112-2

**Published:** 2016-03-25

**Authors:** Christian Klümper, Jörg Neunzehn, Ute Wegmann, Benjamin Kruppke, Ulrich Joos, Hans Peter Wiesmann

**Affiliations:** Institute of Materials Science, Technical University of Dresden, Chair for Biomaterials, Budapester Strasse 27, D-01069 Dresden, Germany; International Medical College of Münster, Schorlemerstraße 26, D-48143 Münster, Germany; Gymnasium St. Christophorus, Kardinal-von-Galen-Str. 1, 59368 Werne, Germany

**Keywords:** Internet-based blended learning, e-learning, Online live lectures, Biomedical education, Pre-university science education, Undergraduate study

## Abstract

**Background:**

Biomedical science, especially biomaterials, is an expanding field in medicine. Universities are being challenged to gain the best students for a later academic career. Pre-university assessment of pupils has become crucial to reach this aim. Blended learning is an emerging paradigm for science education even though it has not yet been rigorously assessed, especially in the pupil/undergraduate situation. The aim of the study was to develop and preliminarily test a blended-learning system in biomedicine for university applicants.

**Methods:**

An internet-based blended-learning module in material science was developed in close collaboration between a university (Biomaterials Department, Dresden TU), a German Gymnasium and an internationally oriented medical college (IMC®, Münster). Forty pre-university students were taught by this learning module composed of school education and internet-based knowledge transfer and involved in the evaluation of the utility of this learning tool. Finally, the students took first-year university examinations in order to evaluate the success of this kind of education.

**Results:**

The internet-based blended-learning module as a combination of e-learning tutorials and live online lectures which was applied in phase 3 of this study was developed on the basis of the findings of both pre-university studies. The results of the learning behavior regarding the number of invokes and the dwell time of the individual pages of the pre-university learning material, the results of the online evaluation and the results of the pre-phase examination were successively used to optimize the next phase. At the end of the pre-university learning, seven of eight participants were able to pass the first-year university examination followed by nationally accepted credit award.

**Conclusion:**

Internet-based blended-learning module proved to be suitable to prepare students for biomedical university education while also giving them the possibility to assess their qualifications for studying biomedicine and subsequent scientific careers. Moreover, the module can help universities to find the best students.

## Background

Biomedicine and especially biomaterials are growing fields in medicine, dentistry and applied natural sciences. The growing need for qualified doctors and engineers in the clinical and scientific fields can barely be covered.

Universities are faced with the task of gaining the best students for these fields of research and possibly even for subsequent scientific careers.

However, many prospective students are not aware of the syllabus profile and the requirements in specific professional fields (such as biomedicine) and the related challenges and opportunities.

For this reason, a significant number of students give up their studies at an early stage, especially in Science, Tech, Engineering, Mathematics, the so-called STEM fields.

Being overwhelmed by university requirements, lack of knowledge of the curriculum and the corresponding professionas well as a completely new and never previousy experienced culture of learning at a university are given as reasons for discontinuation of studies [1, 2].

Against this background it is important that responsible institutions (high schools, colleges and universities) develop new concepts or revise their existing admissions policies. One way of acieving this would be to enrol pupils on pre-admission courses. However, this is generally associated with presence at the university.

These places and the time limitations of pre-student teaching are big hurdles if equal and fair conditions to all potential students are to be offered, especially if the project is considered as a global issue. To resolve this problem, there has been consensus among social institutions, schools and universities to open up new ways to optimize the transition from school to university [3].

The aim of this study was to bridge this gap by the development of a pre-student learning concept meeting university-relevant requirements of a specific study program (biomaterials). In addition, the relevance of this learning concept was to be tested. For this, it was important to include scientific and academic content and university methods of knowledge transfer in our learning project.

For this purpose, the Institute of Materials Science of the Technical University (TU) of Dresden - together with the German St. Christophorus Gymnasium of Werne and the International Medical College (IMC®) of the MIB GmbH of Münster - have implemented the lecture “Materials Science 1” of the diploma program Material Science as an internet-based blended-learning concept comprising four hours per week as an alternative to traditional classroom pre-study.

## Methods

This specific pre-university study was designed as a three-phase construct with each phase including the development of a learning theory model and its testing. A total of 40 students (15 males, 25 females) participated in the study.

The performed study was conducted in the regular school and university system. Only standard evaluation methods were used. Students participated either in the context of school projects or for personal further qualification (graduate studies). The evaluations were conducted anonymously. For this study ethics approval was not required from the Technical University of Dresden. The students were explained that the participation in the study was voluntary, there was guarantee of confidentiality and anonymity and that non-participation would not cause them any harm. They could also choose to withdraw from the study at any time without giving any reason.

In the first phase specific requirements of the Materials course of the “Materials Science” diploma program at the Dresden TU were taken as the substantive basis and implemented as an e-learning model specially for pupils.

The content was given to sixteen students of the 12^th^ grade and tested in under exam conditions. Also in phase 1, it was investigated whether the developed learning concept (contents and method of transfer) was suitable for basic Material Science knowledge transfer in a pre-study course and how students acquire academic contents via internet-based blended-learning.

The technical implementation was based on the e-learning platform “e-med” of the IMC®, which has been successfully used for 12 years in the field of postgraduate master programs in international university continuing education [4].

The second phase was an extension of the first phase. Taking into account the results of the evaluation of phase 1, further contents of the first university semester were imparted as teaching content and, in addition, other university methods of learning and teaching (namely the learning letter “Smart Materials”) were added to the pre-university education. In particular, it was investigated whether the e-learning contents enabled the pupils to handle successfully a task from the first semester of the Materials Science program at university level.

In the third phase, the established learning methods of phase 2 were complemented by the addition of online lectures, which were broadcast live, and followed by discussions as is usually done at universities. The live online lectures were given by professors in the early evening exclusively for pre-students working at home. In addition, the lectures were recorded and thus always available to the students on the e-learning platform on the web.

The third phase of this internet-based blended-learning module concluded with a written examination with required attendance at the Technical University of Dresden, which was conducted for the pre-students at the same time as fro regular students and under identical conditions.

The final learning concept was accepted by the Technical University of Dresden as relevant preparation for obtaining credits. Passing the examination led to a university-wide accepted certificate.

**Fig. 1:**
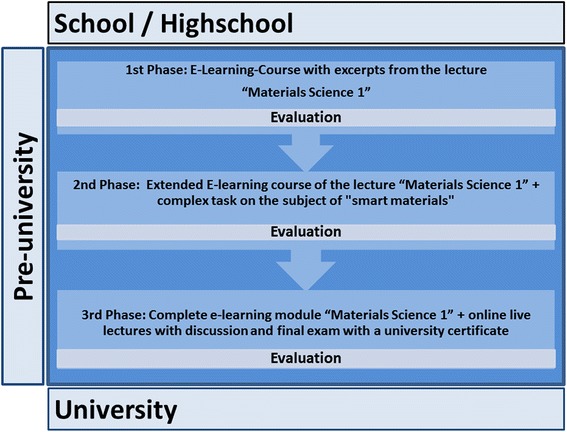
Phases of the learning model

During all phases of the learning model (Fig. 1) the contents were adapted to the lerners’level of knowledge.

In this context, complex subjects were arranged for the study group in a way suitable for pupils in order to achieve manageability and comprehensibility. The contents were understandable, well-structured and complemented by extensive schematic illustrations and video sequences thus making the learning material more diversified [5–7].

The e-learning-lectures included exercises as online tests either in the form of multiple-choice questions or as exercises with self-check and correct solution. The development of the exercises was oriented toward the learning objective to enable the pre-students to evaluate their own learning progress [5, 8, 9].

After completion of the internet-based blended-learning module in the first two phases, learning success was tested in an online examination.

In phase 1, the examination consisted of 10 multiple-choice questions and in phase 2 of 15 different types of questions, related exclusively to reproductive knowledge of the lessons. The examinations were without time limits.

Each of the three concept phases and the examinations were evaluated regarding educational relevance by the students.

Parameters of evaluation were both quality of knowledge contents and presentation and comprehensibility.

Other parameters were related to the working behavior within the work groups with respect to the subject “Smart Materials” and to the assessment of the concept as a whole. In addition, aspects of the internet-based learning behavior (click statistics) and communication patterns were evaluated. The evaluation was based on the German school grade system from 1 (very good) to 6 (unsatisfactory). Firstly, the e-learning component of the project, secondly practical aspects, and thirdly, questions about the overall concept were evaluated.

## Results

### Internet-based blended-learning behavior

**Fig. 2:**
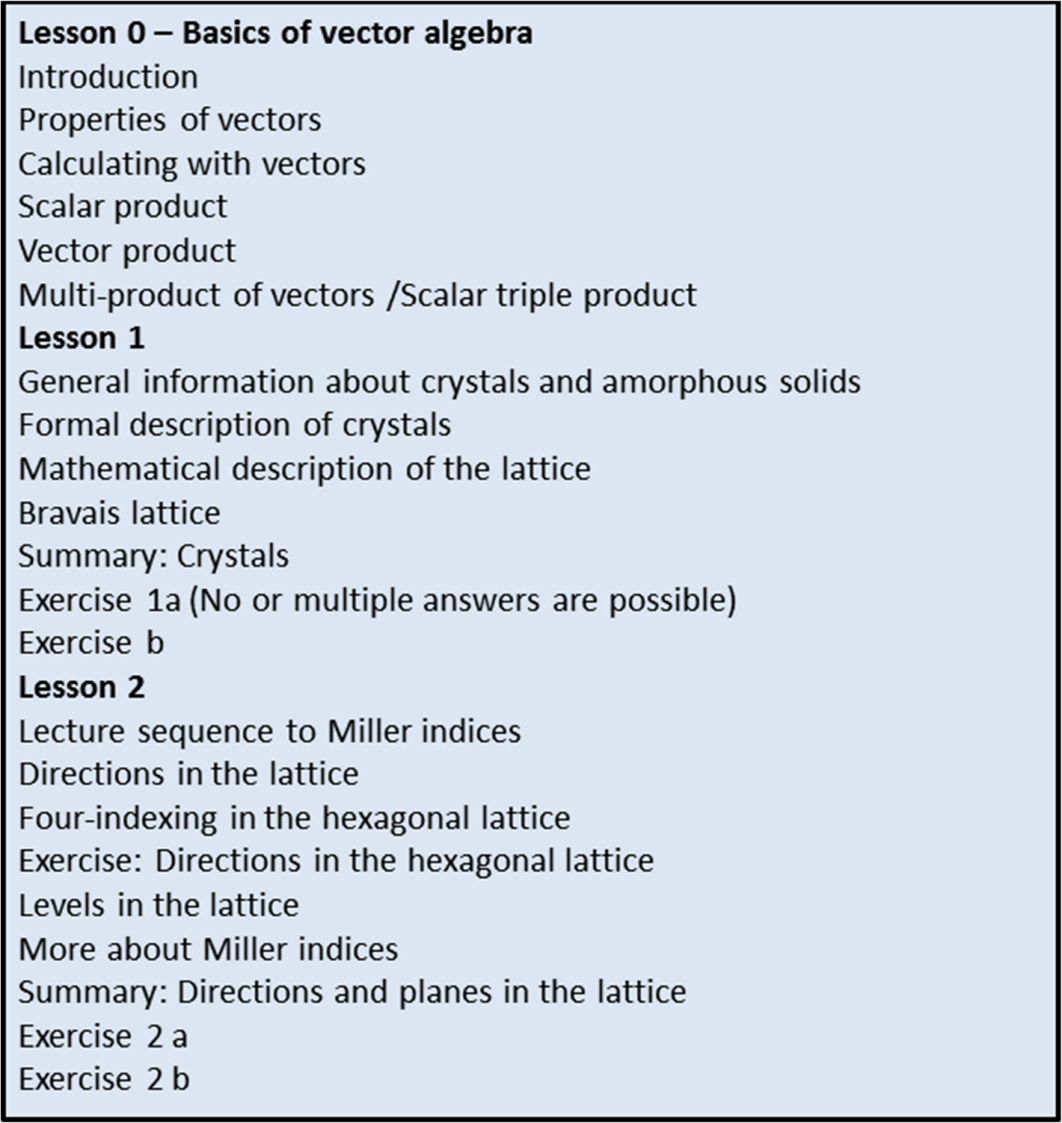
Three lessons of phase 1

In the first phase, the pre-university learning content of the lecture Material Science was divided into 23 learning units, which in turn were allocated to three lessons (Fig. 2). The content was clearly displayed in the content area and included 23 web sites. Moreover, the click statistics also took into account other functionalities, such as the notes page and solution page of the exercises.

**Fig. 3:**
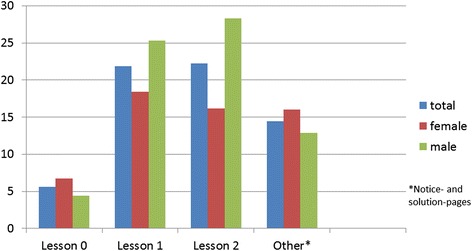
Average number of page views – phase 1

**Fig. 4:**
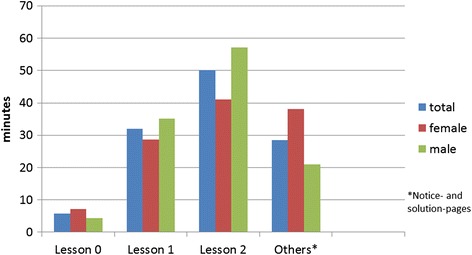
Dwell time in minutes - phase 1

Considering the separate lessons, it was found that the female pupils visited the web pages of lesson 0 more often and with a longer dwell time than the male pupils (Figs. 3 and 4). In total, the female pupils invoked the pages of lesson 1 less often and with a lower dwell time than the male pupils (Figs. 3 and 4). The web sites of Lesson 2 were clicked less often and with lower dwell time by the female pupils than by the male pupils (Figs. 3 and 4). In this lesson, a video sequence “Miller Indices” was integrated. It was invoked with a longer dwell time by the male pupils than by the female pupils.

**Fig. 5:**
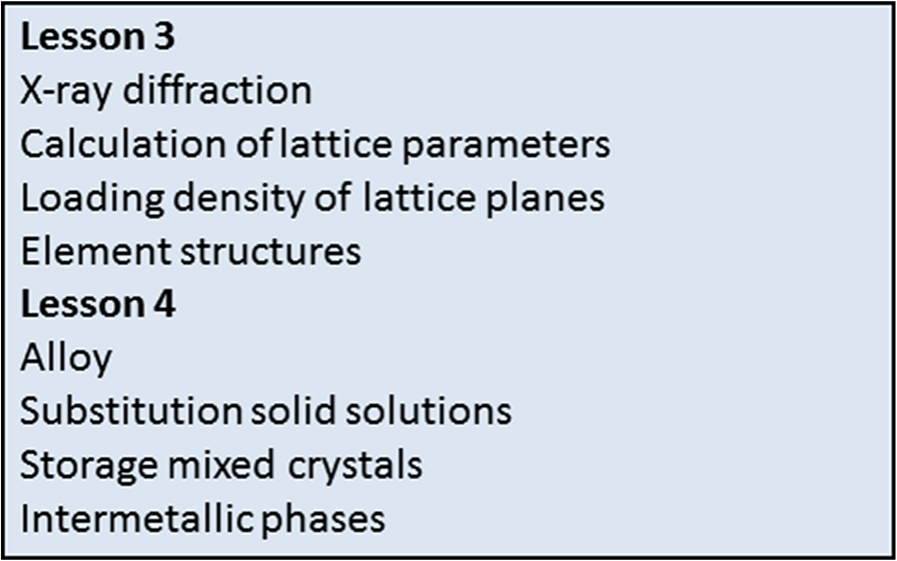
Additional lessons – phase 2

The second phase is an extension of the first phase. The three lessons of the first phase were supplemented by two further lessons to a total of 31 learning units (Fig. 5).

**Fig. 6:**
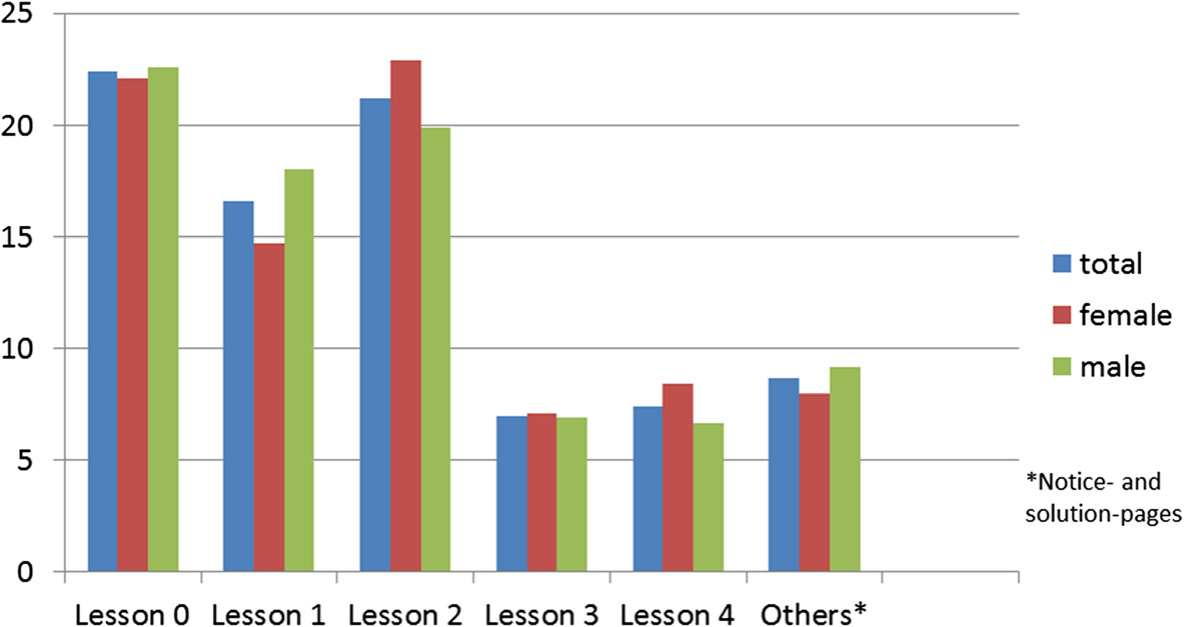
Average number of page views – phase 2

**Fig. 7:**
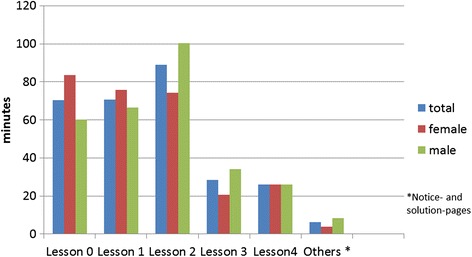
Dwell time in minutes - phase 2

The web pages of lesson 0 were clicked on average nearly identical by female and male pupils whereby the female pupils devoted more time on average (Figs. 6 and 7). In contrast to the first phase, where the content was already known to the pupils from their classes, the content of the lesson 0 was not known to the pupils of the second phase from the classes. In total, the female pupils invoked the pages of lesson 1 less often with a longer dwell time than the male pupils (Figs. 6 and 7). The web sites of lesson 2 were clicked less often with a longer dwell time by the male pupils than by the female pupils (Figs. 6 and 7). The video sequence “Miller Indices” was invoked more frequently, with a longer dwell time by the pupils of the second phase than by the pupils of the first phase. Compared with lessons 0, 1 and 2 the web pages of lesson 3 were invoked on average less often and with a shorter dwell time. In total the male pupils stayed longer on the pages than the female pupils with a nearly identical number of invokes (Figs. 6 and 7). The web sites of lesson 4 were clicked as lesson 3 and in contrast to the first 3 lessons less often and with a shorter dwell time. In total, the female and male pupils stayed for nearly the same length of time (Figs. 6 and 7).

### Pre-phase examination

This pre-university exam was rated with school grades. All students passed the exam. On average, the pupils of the first phase achieved grade “satisfactory”, of the second phase the grade “sufficiently”.

### University examination with credit award

The university test was written by the pre-students at the same time respectively under identical conditions but outside of the university. Of the eight students who took the examination seven passed. Their grades were as follows (Table 1):

**Table 1:** Results of the university exam

Grade	2.7	3.0	3.3	3.7	4.0	5.0
Number of students	1	1	3	1	1	1

### Results of the evaluation of the learning model

Of the 32 students of phases 1 and 2, all took part in the online evaluation. The evaluation was carried out anonymously.

The comprehensibility and the designing of the web-based content, as well as the navigation within the learning content, were evaluated positively by the pupils. The exchange and discussion among the students was very important. This confirms that an online course requires adequate communication facilities for both the pupils among themselves and between pupils and the professor or lecturer.

The scope of the content in terms of available time was considered to be extensive. The overall gain in knowledge and the total interest were rated highly. The concept was considered as stimulating, especially the included video sequence which was recognized as a good didactic tool, and the insights of this were assessed highly. The online exam was evaluated as hard and received the lowest score.

All 16 students of the second phase evaluated the aims of the problem to be solved in the practical part as very understandable and the material provided for the experiments as very positive. They pointed out that style of the presentation was very nicely done. The preparation of the practical part through the e-learning theoretical part was assessed as satisfactory.

The whole concept was evaluated by the 32 pupils with the grade “good”. Overall the pupils have got an overview and an idea of the knowledge transfer at a university.

Of the students, 14 would be happy to participate again in a blended-learning seminar whilst 18 were not sure about it.

## Discussion

Various institutions emphasize that the main challenge of the education system is the transition from school to university. There are already different approaches and offers especially in the field of medicine like the pre med school entry courses for aspiring medical students in the UK and US or the one year pre course at the Medical Universities in Hungary. Finally the problem of optimizing the transition interactions has not yet been finally resolved.

On the one hand, there is the question of how universities can gain the best pupils as well as how univerisities become acknowledged centres of excellence on the other hand, how pupils can best find out whether the envisaged study is appropriate for them.

Our internet-based blended-learning module proved to be a successful system of pre-university student education which also led to nationally raccepted university certificate. In addition, the internet-based blended-learning module turned out to be a helpful tool in closing the gap between school and university education. Widespread establishment of such a model would facilitate mastering of formal university requirements at an early stage. This is important for several reasons:

1. Pupils (pre-students) can self-test their ability to meet the specific requirements of study programs.
2. Periods of study can be reduced accordingly.
3. The universities are enabled to find and recruit exceptionally talented pupils.
4. Independent of their place of residence pupils, can obtain credits, usually without significant costs.
5. Pupils do not miss school lessons.

In the US, the scientific studies regarding Early Study Programs have been conducted since its introduction. Brody and Stanley (1991, 2005) and Stanley (1996, 1997 and 2005) carried out a systematic evaluation of academic success in terms of the duration of studies andacademic achievements of pre-students which were based on their previous studiis. They concluded that pre-students complete regular study programs very successfully and often in shorter study periods. Moreover, they also achieve further academic successes [10–14].

In Austria the project “Pre-students at the Universities” of “özbf” has been established for more than 10 years. Their studies showed that early contact with the college lowered the inhibition threshold in students from educationally disadvantaged and economically less privileged families in pursuing university education [15]. Further cooperation between schools and universities were encouraged [16].

In Germany, since 2004, almost all universities have offered the opportunity for talented pre-students to study university course contents, to acquire university performance records and also, if possible, to obtain university degrees [17]. It was shown that pre-study programs were assessed as very positive by all those involved and highly accepted by the students, and that universities offer large choices of studies [18–20]. However, participation in pre-studies proved to be very time-consuming for many pupils, requiring a high commitment and proximity of residence to the university as there are usually attendance courses.

This is where the advantage of our internet-based blended-learning module comes into the play, allowing studies to be independent of the place of residence and thus accessible to considerably more interested pupils. In addition, it is much easier to pupils to complete the programme because of its compatibility with everyday school life in comparison to campus programs.

The positive opportunities arising from an early approach to university education and our applied internet-based blended-learning concept were supported by first evaluations of the Junior Studies at the University of Rostock, Germany. These studies showed that the pre-students valued the blended-learning concept in form of video lectures. It was found that pupils who chose the Junior Studies are primarily interested in natural science and very talented. Moreover, it became apparent that only rarely were pre-studies given up and that the University of Rostock has become more attractive place for subsequent study [21, 22].

The comparison of classical lectures and blended-learning methods regarding learning behavior of students as well as their satisfaction carried out by the Public Health School of the Tehran University of Medical Science showed that blended-learning methods can lead to better learning ability of students, enrich traditional teaching and sometimes even function as a teaching alternative, increasing knowledge, satisfaction and attentiveness of students [23].

In 2010, a postgraduate online master program in “Advanced Oncology” was established at the Medical Faculty of the University of Ulm. It was found that e-learning in medical biometrics with appropriate extensions to blended- learning approach was possible [24].

The internet-based blended-learning module as a combination of e-learning tutorials and live online lectures which was applied in phase 3 of this study was developed on the basis of the findings of both pre-university studies. The results of the learning behavior regarding the number of invokes and the dwell time of the individual pages of the pre-university learning material, the results of the online evaluation and the results of the pre-phase examination were successively used to optimize the next phase. It is a remarkable result that seven of the eight students of phase 3 successfully absolved the final exam in addition to the classroom.

The internet-based blended-learning module as a combination of e-learning tutorials and live online lectures as applied in phase 3 of this study can be beneficial for academic learning as confirmed by successful completion of examinations and related university certification.

## Conclusion

The pre-university internet-based blended-learning concept allows location-independent studying with award of university-related certificates for pre-students. This approach initially confined to biomedicine should also be developed, evaluated and established in other fields, aiming to bridge the gap between school and university education.

## Abbreviations

IMC®: International Medical Colllege
özbf: Österreichische Zentrum für Begabtenförderung und Begabungsforschung
STEM: Science, Technology, Engineering, and Mathematics
